# Symptomatic Floor-of-Mouth Swelling with Neck Extension in a 14-Year-Old Girl

**DOI:** 10.1155/2014/831923

**Published:** 2014-12-03

**Authors:** Kristin Dayton, Matthew F. Ryan

**Affiliations:** ^1^Department of Pediatrics, University of Florida, Gainesville, FL 32610, USA; ^2^Department of Emergency Medicine, University of Florida, Gainesville, FL 32610, USA

## Abstract

A plunging ranula is a soft-tissue mass stemming from a mucous extravasation cyst of the sublingual gland which can herniate through the mylohyoid muscle. We describe a case in which a 14-year-old girl presented with a rapidly expanding mass on the floor of her mouth affecting her ability to swallow and speak and causing tracheal compression. The patient was initially managed conservatively with antibiotics and steroids; however, the mass continued to expand necessitating emergent bedside incision and drainage and subsequent surgical intervention. The pathophysiology and management options for ranulas are also discussed herein.

## 1. Introduction

A plunging ranula is a soft-tissue mass stemming from a mucous extravasation cyst of the sublingual gland which can significantly expand and thus, via mass effect, herniate through the mylohyoid muscle [1, 2]. Ranulas have a prevalence of about 0.2 in 1000 but those that cause tracheal compression and airway compromise are even more rare [2]. We describe a case in which a 14-year-old girl presented with a rapidly expanding mass on the floor of her mouth which was significantly affecting her ability to swallow and speak and concomitantly causing tracheal compression. Management of a ranula is varied and often surgical removal of the sublingual gland is needed for successful resolution [1]. This case is important because it underscores how seemingly benign oral masses especially in children can have worrisome consequences. Moreover, it underscores the need for the physician to ensure continued monitoring for patient safety and to avoid untoward complications even in previously healthy children with no significant medical history. Details of the case are presented along with a review of the acute and long-term management of oral ranulas.

## 2. Case Presentation

A 14-year-old otherwise healthy Caucasian girl presented to the pediatric emergency department complaining of a swelling under her tongue. Although the swelling started insidiously approximately four weeks prior to presentation, the mass had expanded over a three-hour period. The patient was seen by her pediatrician a few weeks ago when the lesion was smaller; she was treated at that time with a course of oral antibiotics (the patient's mother could not recall which antibiotic was prescribed) for possible infection. Nevertheless, the lesion did not improve and the patient was referred to an otolaryngologist for further evaluation. However, because the lesion expanded rapidly, the patient presented acutely to the emergency department for evaluation.

Upon arrival, the patient appeared anxious but in no distress. Prior to arrival, the patient reported she was in her normal state of health and ate a normal dinner. Shortly thereafter she noticed an increased fullness under her tongue in which the ranula expanded from about 0.5 cm in diameter to its current state of approximately 5 cm in diameter; moreover, the expanding mass was impairing her ability to swallow and speak. The patient noted the mass was painful which worsened when she tried to open her mouth.

The patient denied fever, chills, weight loss, diet changes, cold and flu-like symptoms, shortness of breath, chest or neck pain, or recent trauma to the area. The patient's past medical, surgical, family, and social history were noncontributory. The patient had stable vital signs and she was afebrile with a pulse oximetry reading of 99% in room air. On exam, the patient was noted to have a large bluish intraoral swelling under the tongue which was most prominent on the right side and extended to the left side of the floor of her mouth. Her tongue was elevated superiorly due of the size of the lesion and she has difficulty speaking clearly. There was swelling noted in the submandibular area which was fluctuant and tender to palpation. There was no erythema or induration of the neck and no cervical lymphadenopathy was appreciated.

**Figure 1:**
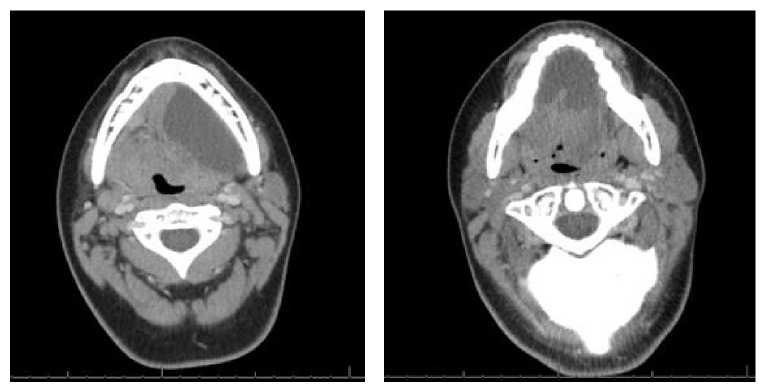
CT scan with IV contrast revealing a fluid filled, unilocular sac with extension across the midline and into the submandibular space.

**Figure 2:**
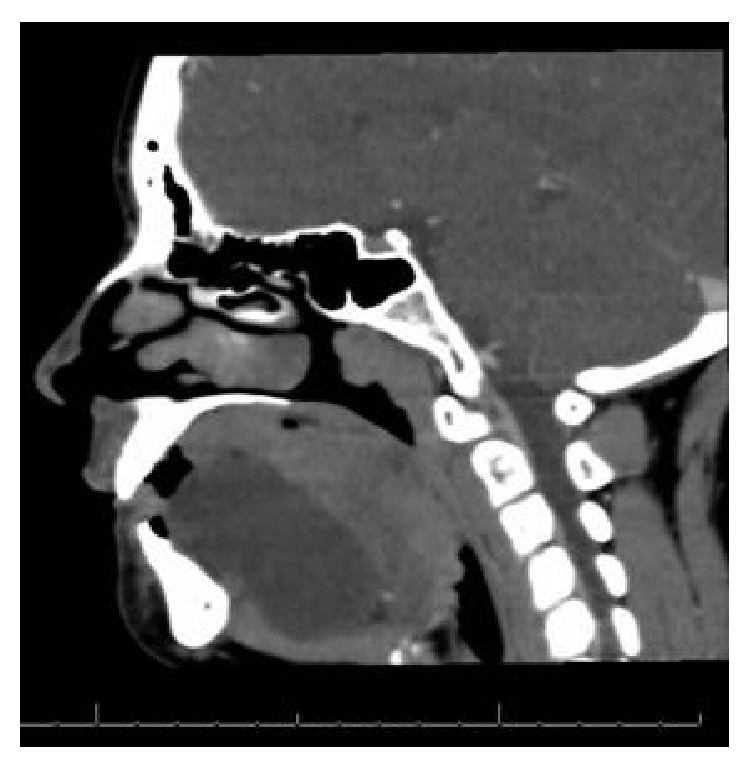
CT image demonstrating tracheal compression secondary to a large expanding ranula.

A computed tomography scan (CT) of the patient's face and neck with intravenous (IV) contrast revealed a large fluid collection in the right sublingual space (Figure 1) extending through the right mylohyoid muscle and crossing midline anterior to the left submandibular space measuring 7.1 cm × 3.3 cm × 2.8 cm (66 cm^3^). There was no enhancement with IV contrast; however compression of the trachea due to mass effect was evident (Figure 2).

The patient was given methylprednisolone 125 mg IV, morphine 2 mg IV, and clindamycin 900 mg IV and admitted to the hospital out of concern for potential airway compromise as well as inability to maintain oral intake due to progressively worsening swelling of the oral mass. While still in the emergency department, the lesion continued to swell and the decision was made to drain the ranula at bedside. After initial needle aspiration, an incision and drainage was performed which produced approximately 80 mL of viscous fluid consistent in appearance with saliva. Fluid culture ultimately grew* Haemophilus influenzae*. The patient was started on a 7-day course of oral clindamycin and discharged home the next day without further sequelae.

The patient was later seen by an otolaryngologist who initially opted for conservative management; however, the lesion has since recurred and surgery or sclerotherapy is planned.

## 3. Discussion

A plunging ranula, also known as a cervical or diving ranula, is a rare clinical entity. The name ranula derives from its similar appearance to the air sacs in a frog's neck [1] where the Latin name* rana* means frog. A ranula is an extravasated mucocele derived from the sublingual gland which lacks a proper capsule. Rupture of the main ducts or acini of the sublingual gland occurs due to obstruction which can lead to the formation of a ranula. The extravasated mucus triggers a localized inflammatory response and becomes enveloped in fibrous granulation tissue. In general, ranulas continue to enlarge because the sublingual gland is a constitutive secretor of mucus. Ranulas can be limited to the intraoral region or they can expand and herniate through or around the mylohyoid muscle which serves as an anatomical barrier between the sublingual and submandibular regions. When a ranula extends through or around the mylohyoid muscle, it is termed as a plunging ranula and often presents with swelling in the submandibular or cervical areas [2].

The typical clinical presentation of a ranula is a painless, progressive, and fluctuant floor-of-mouth or neck swelling that can recur over time [3]. The intraoral component usually has a bluish color and is typically unilateral, although bilateral swelling can occur [4]. It is rare for ranulas to present with pain and acute enlargement as described herein [5] and airway involvement is especially rare. The differential diagnosis for a floor-of-mouth ranula includes an abscess, dermoid cyst, or rarely malignancy. When the neck is involved thyroglossal cyst, branchial cleft cyst, cystic hygroma, hemangioma, lymphangioma, or acute inflammatory lymphadenopathy should be considered in the differential diagnoses [4–6]. A congenital predisposition to plunging ranulas may exist especially as they are more common in Maori and Pacific Island ethnic groups as well as other populations of Asian descent [7]. Ranulas develop slowly and typically present in the second and third decades of life [8].

The diagnosis of oral ranula can be made clinically. However, most providers advocate for imaging studies if a plunging ranula is present, especially if there is cervical swelling and no intraoral component [9]. Multiple studies have demonstrated that high resolution ultrasound is a useful clinical tool when the diagnosis is uncertain [3, 9, 10]. Advanced imaging such as CT and magnetic resonance imaging (MRI) can delineate the anatomy and help with surgical planning; note that advanced imaging entails higher costs and CT has the added risk of high radiation exposure [9] which may outweigh the benefit of its need. Assay of the fluid aspirated from ranulas will reveal high amylase and protein levels consistent with the presence of saliva [11, 12].

Acute management involves supportive care. In our case, the patient had difficulty swallowing and speaking as well as tracheal compression evident on imaging (Figure 2) which warranted admission for observation. She denied any shortness of breath during this time. Fluid may be drained from ranulas to temporarily alleviate the swelling, but most times it will reaccumulate. It is rare for airway compromise to be present, but if the patient presents with respiratory distress, it is crucial to secure an airway as the swelling could continue to worsen resulting in airway compression and compromise [5]. Patients should be evaluated by a specialist in otorhinolaryngology or dentistry/oral surgery as soon as possible for definitive management. While specific surgical management practices vary, most sources agree that excision of the sublingual salivary gland is the most effective treatment to minimize recurrence [11, 12].

## 4. Conclusions

A plunging ranula is a rare clinical entity; however, it should be considered in the differential diagnosis in a child, especially in the second decade of life, who presents with progressive, painless, and fluctuant neck swelling. Ultrasound and fluid aspirate analysis can be effective for a clinical diagnosis and surgical planning. Advanced imaging is usually not required and can add unnecessary healthcare costs and radiation exposure. Definitive management of ranulas usually involves surgical excision of the sublingual gland. Precautions regarding any expanding neck mass should always entail continued observation and airway protection.
